# Cannabidiol and Tetrahydrocannabinol Use in Parkinson’s Disease: An Observational Pilot Study

**DOI:** 10.7759/cureus.42391

**Published:** 2023-07-24

**Authors:** Amber Sousa, Joanne DiFrancisco-Donoghue

**Affiliations:** 1 Osteopathic Manipulative Medicine, New York Institute of Technology College of Osteopathic Medicine, Old Westbury, USA

**Keywords:** tetrahydrocannabinol, cannabidiol, complementary and alternative medicines, cognition, neuropsychology, cannabinoids, parkinson's disease

## Abstract

Background: There is a need for more research examining the use of cannabis, tetrahydrocannabinol (THC), and cannabidiol (CBD) products in people with Parkinson’s disease (PD), especially given the recent increase in the use of these products.

Objectives: Given the recent increase in over-the-counter CBD use as well as the prescription of medical cannabis by treating physicians, the utilization method, effects on motor and non-motor symptoms, side effects, and attitude toward cannabis use were examined in a naturalistic sample of patients with PD.

Methods: A total of 15 individuals with PD, eight of whom were prescribed CBD/THC treatment and seven who were not taking any CBD/THC product, were assessed cross-sectionally. Participants completed structured neuropsychological testing, motor assessment, and questionnaires regarding mood, subjective cognition, and symptom levels. T-tests were completed for quantitative measures and descriptive data were examined and described. Due to the small sample size, Shapiro-Wilk tests for normality were utilized and Mann-Whitney U analyses were completed when appropriate.

Results: We found a wide range of prescribed products and methods as well as variability in perceived benefits and untoward effects, even in our small sample. Individuals with PD who were taking a CBD/THC product had lower global cognition scores on the Montreal Cognitive Assessment (MoCA) but no detectable differences among more specific neuropsychological measures. They also had more non-motor symptoms of PD but no differences in motor symptom levels. Qualitatively, some participants with PD who were taking CBD/THC reported improved pain levels, sleep, and reductions in anxiety. A few negative effects were endorsed, including sleepiness, concentration difficulties, and forgetfulness.

Conclusion: CBD/THC utilization in PD is varied. In our small sample, individuals who utilized the treatment had lower MoCA scores, more non-motor symptoms, and descriptively reported improvements in sleep, anxiety, and pain, and had side effects of sleepiness and cognitive difficulty. Future studies should focus on clinical trials with standardized CBD/THC methods of use.

## Introduction

Parkinson’s disease (PD) is a progressive neurodegenerative disorder that was first described by Dr. James Parkinson in 1817 [1]. Its prevalence increases with age and was found to be 572 per 100,000 in people over the age of 45 years in North America [2]. The disease can involve both motor symptoms, including tremor, rigidity, bradykinesia, and postural instability, as well as non-motor symptoms, including sleep disturbance, depression, cognitive deficits, and neuropsychiatric symptoms. Currently, conventional treatments for PD include dopamine precursors, dopamine agonists, monoamine oxidase inhibitors, and catechol-O-methyltransferase inhibitors. Various other treatments are utilized for the wide range of symptoms that patients with PD encounter [3].

Although human use of cannabis dates back hundreds of years, evidence for efficacy, especially for specific disorders, remains elusive. There remains a general lack of controlled trials due to factors including varying legality, non-uniformity in drug components and doses, side effects, and stigma [4]. Despite these challenges, given cannabis’ long history and breadth of use, much literature exists regarding its potential methods of utilization (see Brunetti et al. (2020) [5] for a review and guide for prescribing doctors). However, tetrahydrocannabinol (THC), cannabis, and cannabidiol (CBD) are marketed for a very wide range of conditions, including a host of medical, psychiatric, and neurological disorders. Specifically for PD, there seems to be a need for guidance in clinical practice as data suggest a lack of consensus among practitioners, even at the National Parkinson Foundation Center of Excellence [6].

Controlled trials of cannabis or CBD in the area of movement disorders or PD are sparse and results of available studies have been mixed and difficult to compare. A recent systematic review of clinical studies concluded that cannabinoids did offer some potential therapeutic effects for both motor and non-motor symptoms [7].

In a study examining the acute effects of smoked cannabis, 22 patients with PD experienced significant improvements in tremor, rigidity, and bradykinesia 30 minutes after cannabis use [8]. In a trial of 21 patients assigned to either placebo, CBD 75 mg, and CBD 300 mg, there we no significant differences in Unified Parkinson's Disease Rating Scale (UPDRS) scores, but an improved quality of life in the 300 mg group as measured by the Parkinson’s Disease Questionnaire (PDQ-39) [9]. In a survey regarding cannabis use in PD patients, 25% reported utilizing cannabis, and 45% of those individuals noted improvements in tremors, bradykinesia, muscle rigidity, and dyskinesia, while only 5% reported a worsening of symptoms. In another study, survey responders reported significant improvements in mood, memory, and fatigue [10]. Finally, a systematic review and meta-analysis concluded that while there is no compelling evidence for the use of cannabis in PD, there are potential treatment effects for PD-related tremors, anxiety, and pain, and improvement of sleep quality and quality of life [11].

In this small study of CBD and THC use in PD, we examine objective and subjective cognitive function, associated symptoms of disease, patterns of use, and subjective assessment of outcomes. We report quantitative data for objective measures and qualitative data for the results of our questionnaire on the utilization of and experiences with CBD/THC in PD.

## Materials and methods

Participants

Participants were recruited between June 1, 2019, and March 11, 2020, from the Adele Smithers Parkinson’s Disease Treatment Center in Old Westbury, NY, and from local events. Data were accessed for analysis from May 14, 2020, to July 14, 2020. Inclusion criteria were age above 18 years, diagnosis of PD by a neurologist, and current use of one or more forms of CBD/THC. This study was approved by the New York Institute of Technology Institutional Review Board under study number BHS-1480 and written informed consent was obtained from each participant. Each participant completed a demographic form, a survey of cannabis/CBD/THC use, and a battery of neuropsychological cognitive measures with a licensed neuropsychologist, including the Montreal Cognitive Assessment (MoCA) [12] to measure global cognition. This is a pencil and paper test that takes about 10 minutes and is completed face-to-face with participants but asking questions verbally, presenting images on paper, and having the participant use a pencil for parts of the measure. Other pencil and paper tests, including measures of attention and executive functions specifically, are the digit span test [13], the Trail Making Test A and B [14], language tests, including the Boston Naming Test [15], semantic and phonemic fluency [16], memory tests, including the Hopkins Verbal Learning Test-Revised (HVLT-R) [17], and psychomotor test, including the nine-hole pegboard dexterity test, which utilized a pegboard and plastic pegs [18]. Researcher-administered observational measures and questionnaires related to symptoms of PD included the Movement Disorder Society-Unified Parkinson's Disease Rating Scale (MDS-UPDRS; part 1: non-motor aspects of experiences of daily living; part 2: motor aspects of experiences of daily living; part 3: motor exam) [19]. Self-reported questionnaires and inventories of motor function, mood, quality of life, and subjective cognition included the Neuro-Qol Upper Extremity Function (UEF) and Lower Extremity Function (LEF), Neuro-QoL Cognitive Function [20], the Epworth Sleepiness Scale (ESS) [21], the Rapid Eye Movement Sleep Behavior Questionnaire (REM SBQ) [22], the Geriatric Depression Scale (GDS) [23], and the Modified Schwab and England Activities of Daily Living (MSEADL) scale [24]. Reducing sources of bias was considered. The MDS-UPDRS was completed by a blinded clinician. All questionnaires and self-report measures were completed by the participants themselves. Neuropsychological tests thought to be more objectively based were completed by the unblinded principal investigator.

Data analysis

We analyzed the quantitative data using IBM SPSS Statistics version 27 (IBM Corp., Armonk, NY) [25]. We employed independent samples t-tests. Given that our primary outcome was global cognition (MoCA) and other outcomes were secondary, no correction for multiplicity was carried out [26]. Due to the small sample size, all outcome variables were tested for normality in distribution using the Shapiro-Wilk method. For all variables that failed Shapiro-Wilk with a p-value below 0.05, variables were rank ordered. Following rank-ordering, Mann-Whitney U tests were carried out.

We aimed to describe the utilization, perceived benefits, and side effects of THC/CBD treatment in a sample of PD patients. We also examined differences between individuals with PD who used CBD/THC compared to those who did not on both subjective and objective cognition, motor function, mood, and other related symptoms of PD.

## Results

Demographics

A total of 15 subjects with a diagnosis of PD completed neuropsychological testing and questionnaires, eight of whom were currently utilizing CBD or cannabis products and seven controls who had never utilized these treatments since their diagnosis with PD. Our entire group of 15 individuals had an average age of 67.5 years (controls = 70.6; CBD/THC = 64.8). The total group was 87.7% male, CBD/THC group was 86.7% male, and the control group was 88.5% male. Years since diagnosis of PD were 4.3 for the control group and 4.9 for those taking CBD/THC.

Cognitive measures

On cognitive testing, global cognitive functioning measured by the MoCA, the average score was significantly higher for the control group than for those taking CBD/THC. For the other neuropsychological measures, no significant differences were found. These included an immediate recall of a list of words, simple auditory attention, working memory, visual scanning speed, mental flexibility, confrontation naming, phonemic fluency, semantic fluency, psychomotor speed, and depression. On the MDS-UPDRS, part 1, which measures the non-motor aspects of experiences of daily living, the CBD/THC group endorsed a significantly higher level of these symptoms than the control group. For parts 2 and 3 of the MDS-UPDRS, no significant differences were found. No significant differences were found for upper extremity function, lower extremity function, subjective cognition, rapid eye movement (REM) sleep behavior disorder symptoms, depression, sleepiness, or activities of daily living. See Table 1 for all outcome measures. Tables 2-4 include collected qualitative data from our CBD/THC use PD questionnaire.

**Table 1:** Outcome measures for groups (control and CBD/THC) Values with asterisks indicate values below 0.05 on the Shapiro-Wilk test for normality. For those variables, rank order was computed and Mann-Whitney U tests were conducted, with results reported in the right-most column. MoCA: Montreal Cognitive Assessment; HVLT-R: Hopkins Verbal Learning Test-Revised; MDS-UPDRS: Movement Disorder Society-Unified Parkinson's Disease Rating Scale; UEF: Upper Extremity Function; LEF: Lower Extremity Function; REM SBQ: Rapid Eye Movement Sleep Behavior Questionnaire; MSEADL: Modified Schwab and England Activities of Daily Living; ESS: Epworth Sleepiness Scale.

Cognitive	Control group, mean (SD) (n = 7)	CBD/THC group, mean (SD) (n = 8)	Total, mean (SD) (n = 15)	Sig (p-value)	Mann-Whitney U Sig (p-value)
MoCA	26.4 (2.5)	23.3 (3.1)	24.7 (3.2)	0.048	
HVLT-R total recall	16.3 (4.5)	17.8 (5.5)	17.1 (4.9)	0.58	
HVLT-R delayed recall	4.7 (4.2)	6.1 (2.9)	5.5 (3.5)	0.45	
Digit Span forward	6.6 (1.3)	7.1 (1.0)	6.9 (1.1)	0.36	
Digit Span backward	4.4 (1.0)	4.5 (1.4)*	4.5 (1.2)	0.91	1.00
Trail Making Test A - time	42.7 (10.3)	39.5 (18.1)*	41.0 (14.6)	0.69	0.34
Trail Making Test B - time	132.4 (68.4)	121.3 (103.0)*	126.5 (85.7)	0.65	0.40
Boston Naming Test	28.1 (3.2)*	28.5 (1.3)*	28.3 (2.3)	0.78	0.54
Phonemic (letter S) fluency	13.6 (5.5)	14.4 (6.6)	14.0 (5.9)	0.80	
Semantic fluency (animals)	16.0 (4.8)	18.5 (7.5)	17.3 (6.3)	0.46	
Nine-Hole Pegboard (dominant hand)	29.8 (3.9)	27.0 (5.7)	28.4 (4.8)	0.39	
Other outcome measures					
Geriatric Depression Scale	2.6 (2.0)	4.1 (3.2)*	3.4 (2.7)	0.29	0.46
MDS-UPDRS Part 1	7 (4.7)	15.6 (8.4)	11.6 (8.0)	0.049	
MDS-UPDRS Part 2	7.8 (4.7)	10.3 (5.5)	9.2 (5.1)	0.41	
MDS-UPDRS Part 3	32.4 (12.9)	26.5 (13.0)	29.0 (12.8)	0.46	
UEF	69.9 (5.8)	67.4 (6.4)	68.5 (6.0)	0.45	
LEF	35.9 (3.8)	35.8 (3.7)	35.8 (3.6)	0.96	
Subjective cognition	115.5 (9.6)	109.6 (22.6)	112.8 (16.1)	0.57	
REM SBQ	4.0 (3.5)	6.0 (3.5)	5.1 (3.5)	0.29	
MSEADL scale	87.1 (9.5)	82.5 (7.1)	84.7 (8.3)	0.30	
ESS	7.9 (3.0)	9.4 (3.9)	8.6 (3.4)	0.41	

**Table 2:** Qualitative data for CBD/THC use and administration For a copy of the questionnaire, including all answer options, please see the Appendix. CBD: cannabidiol; THC: tetrahydrocannabinol.

What type of CBD/cannabis/marijuana/THC product do you use?						
CBD (%)	Cannabis (%)	Combination (%)				
57	14	29				
When did you start taking CBD/THC?						
Within 1 month (%)	Within the past 6 months (%)	Within the past year (%)				
14	43	29				
How were you introduced to CBD/THC?						
Doctor (%)	Friend or family (%)					
43	57					
What method of administration are you prescribed to take your medication and in what form does it come in?						
Oral liquid only (%)	Topical (%)	Multiple forms (vaporized inhalation, oral liquid, tablet, or capsule) (%)				
43	14	43				
On a given day, how many doses of CBD/THC do you take?						
Once per day (%)	Twice per day (%)	Variable times per day (%)				
57	29	14				
How many days per week do you take CBD/THC?						
3-6 times per week (%)	Every day (%)					
57	43					

**Table 3:** Qualitative data for CBD/THC use and symptoms For a copy of the questionnaire, including all answer options, please see the Appendix. CBD: cannabidiol; THC: tetrahydrocannabinol.

Have you noticed a difference in the frequency of tremors on days that you take your CBD/THC?			
Significantly better (%)	Unchanged (%)	Do not have a tremor (%)	
29	43	29	
Have you noticed a change in the amount of pain you experience while taking CBD/THC?			
Significantly better (%)	Slightly better (%)	Unchanged (%)	
71	14	14	
Have you noticed a change in the amount of anxiety you experience while taking CBD/THC?			
Significantly better (%)	Slightly better (%)	Unchanged (%)
14	43	29
Have you noticed a change in your ability to fall asleep while taking CBD/THC?			
Significantly better (%)	Slightly better (%)	Unchanged (%)	
14	29	57	
Have you noticed a change in the level of depressive symptoms while taking CBD/THC?			
Significantly worse (%)	Slightly better (%)	Unchanged (%)	
14	29	57	
Have you noticed a difference in your short-term memory on days that you take your CBD/THC?			
Significantly worse (%)	Slightly worse (%)	Unchanged (%)	
14	43	43	
Have you experienced feelings of “brain fog” or a diminishing ability to process information on days that you are taking your CBD/THC?			
Significantly worse (%)	Slightly better (%)	No brain fog (%)	
14	57	29	
Do you notice a difference in how well-rested you feel in the mornings after taking your CBD/THC medication the night/day before?			
Significantly less (%)	Slightly more (%)	Unchanged (%)	
14	29	71	
Do you notice a difference in your balance after taking your CBD/THC medication?			
Significantly improved (%)	Somewhat improved (%)	Unchanged (%)	
0	14	86	
Do you notice a difference in your “freezing” after taking your CBD/THC medication?			
Somewhat improved (%)	Unchanged (%)	Don’t have freezing (%)	
14	43	43	
Do you notice a difference in your rigidity after taking your CBD/THC medication?			
Significantly improved (%)	Unchanged (%)	No rigidity(%)	
14	57	29	
How has your quality of life changed since taking your CBD/THC medication?			
Significantly improved (%)	Somewhat improved (%)	Unchanged (%)	
14	71	14	

**Table 4:** Open-ended survey question responses CBD: cannabidiol; THC: tetrahydrocannabinol.

Describe some of the positive benefits you have encountered since starting CBD/THC.						
						"better sleep, eases back pain, eases anxiety"						
"less anxiety, better sleep, less tremors"						
"reduced pain in legs"						
"relaxation, decreased anxiety, decreased depression"						
"not exactly positive, just OK, made me somewhat relaxed"						
"improved sleep" “pain relief”						
Describe some of the negative effects you have encountered since starting CBD/THC.						
						"sleepy"						
"concentration loss, forgetfulness"						
Five participants reported "none"						

## Discussion

In this study of a naturalistic sample of people recruited from a clinic and community for PD, we cross-sectionally examined a small group of individuals diagnosed with PD who were either taking a product containing CBD, THC, or both and compared them to a control group naive to such treatment since the onset of their PD illness. We measured objective and subjective cognition, motor function, symptom levels, and mood, and took a survey of CBD/THC use, and perceived effects of CBD/THC treatment.

Those who were prescribed or taking CBD/THC had a significantly lower global cognition score on the MoCA. Other research has found cognitive impairing effects of THC and CBD-THC combination administered by vaporized inhalation. Specifically, Arkell et al. (2019) [27] found reductions in performance on tasks of divided attention, psychomotor speed, and driving ability. These findings highlight the need to monitor for cognitive and functional difficulties in individuals taking CBD/THC. Because our data are cross-sectional, it is also possible that individuals with lower global cognition were more likely to seek out or try non-conventional treatments such as CBD/THC.

Interestingly, all other tests of neuropsychological function, including verbal word-list memory, psychomotor speed, mental flexibility, confrontation naming, and semantic and phonemic fluency, were not significantly different between the two groups. The more sensitive measures of cognition failed to find a difference while the MoCA showed one, which suggests the potential that an unmeasured factor was accounting for global cognitive differences. Our battery did not measure visuospatial construction or orientation to time and place, so these may be areas where MoCA showed a difference that our other cognitive measures did not. A more comprehensive neuropsychological measure battery might elucidate this finding.

Within our measures of mood, motor function, subjective cognition, sleep, and quality of life, the only significant difference was a significantly higher level of non-motor symptoms of PD in the MDS-UPDRS (part 1) in the CBD/THC group. Again, this could reflect either an effect of the CBD/THC treatment or a pre-treatment difference between groups. Speculatively, it is possible that many of the non-motor symptoms of PD are treatment targets for CBD/THC (anxiety, sleep disturbance, and pain).

Qualitatively, some individuals in the CBD/THC group described improvements in pain, depression, and anxiety levels. These findings are in line with preclinical research regarding pain [28]. And in fact, in our sample, 71% of individuals who were utilizing these products reported some reduction in the amount of pain experienced. Rigorous research regarding the treatment of anxiety and depression with cannabinoids is lacking and inconclusive.

No significant differences were found for self-reported motor aspects of experiences of daily living or on the objectively measured motor exam of the MDS-UPDRS. Similarly, a meta-analysis found a lack of evidence for significant treatment effects of cannabis and its derivatives on the motor symptoms of PD [29]. Also consistent with this was our qualitative data, in which only one of the seven participants utilizing CBD/THC indicated a reduction in tremors when taking CBD/THC.

For the qualitative data, in our questionnaire regarding CBD/THC use, we found that even in our small sample of patients, a variety of administration routes and types of products were utilized. This highlights the breadth of the type of usage of these compounds in naturalistic samples, even of individuals in the same geographic treatment area with the same diagnosed neurological condition.

In our study, three individuals noted a subjective improvement in sleep. A recent study also found an improved satisfaction with sleep quality with a dose of 300 milligrams of CBD, but no reduction in REM behavior disorder in PD patients [30]. While five of our participants indicated no noticed negative effects of their CBD/THC treatment, one individual noted increased sleepiness, and one noted increased forgetfulness and concentration loss. While these responses appear to suggest few negative reported side effects, given the known potential psychoactive and cognitive effects of CBD/THC, further investigation is warranted.

Limitations to this research include our small sample size and cross-sectional approach. The small sample size did not allow for meaningful comparisons between variables in people with specific CBD/THC use methods. Because our study was cross-sectional, we were unable to determine whether these differences were due to the treatment or existing group differences, such as individuals with these characteristics being more likely to seek non-conventional CBD/THC treatment. Future work would include randomized clinical trials of CBD/THC treatment protocols and larger sample sizes of patients. This would allow for a more evidence-based approach to the utilization of CBD/THC in people with PD.

## Conclusions

In summary, this cross-sectional investigation of a small group of individuals with PD currently utilizing CBD/THC compared to a group who were not utilizing such agents revealed a lack of standardized treatment protocols when implementing this non-conventional therapy. Additionally, individuals with PD who were receiving CBD/THC had worse global cognition on the MoCA and non-motor symptoms of PD, which could represent either pre-existing differences or effects of CBD/THC. Qualitative responses on a questionnaire revealed a number of perceived benefits, including pain reduction, anxiety reduction, and improved sleep quality. Reported side effects included sleepiness and cognitive difficulties. Future research should include clinical trials with larger sample sizes and control of CBD/THC products and doses.

## Appendices

Survey of CBD/THC therapy in Parkinson’s disease

1. What CBD/cannabis/marijuana/THC product do you use?

a. CBD

b. THC prescribed

c. Combination

d. Other: explain

2. When did you start taking CBD/THC?

a. Within the past month

b. Within the past six months

c. Within the past year

d. Longer than a year: explain

3. How were you introduced to CBD/THC? (Doctor, family member/friend, news article, etc.)

a. Doctor

b. Friend/family

c. Advertisement

4. What method of administration are you prescribed to take your medication and in what form does it come in? (Oil for under your tongue, pills taken orally, vaporized CBD/THC, etc.)

a. Oral liquid

b. Vaporized inhalation

c. Smoked

d. Tablet/capsule

e. Edible form

f. Other/combination: explain

5. On a given day, how many doses of CBD/THC do you take? Describe your dosing regimen below (e.g., twice a day: once when waking up, the other before bed).

a. Once per day

b. Twice per day

c. Three times per day

d. Other: explain

6. How many days per week do you take CBD/THC?

a. Less than once per week

b. Once or twice per week

c. Three to six times per week

d. Every day

7. Below, in your own words, describe some of the positive benefits you have encountered since starting CBD/THC, if any. __________________________________________________________________________________________________________________________________________________________________________________________________________________________________________

8. Below, in your own words, describe some of the negative effects you have encountered since starting CBD/THC, if any. __________________________________________________________________________________________________________________________________________________________________________________________________________________________________________

The below questions ask specific questions about how this medication helps or does not help you:

9. Have you noticed a difference in the frequency of tremors on days that you take your CBD/THC?

1. Tremor is significantly better

2. Tremor is slightly better

3. Tremor present is not changed

4. Tremor present is slightly worse

5. Tremor is significantly worse

10. Have you noticed a change in the amount of pain you experience while taking CBD/THC?

1. Pain is significantly better

2. Pain is slightly better

3. Pain is unchanged

4. Pain is slightly worse

5. Pain is significantly worse

11. Have you noticed a change in the amount of anxiety you experience while taking CBD/THC?

1. Anxiety is significantly better

2. Anxiety is slightly better

3. Anxiety is unchanged

4. Anxiety is slightly worse

5. Anxiety is significantly worse

12. Have you noticed a change in your ability to fall asleep while taking CBD/THC?

1. Falling asleep is significantly easier

2. Falling asleep is slightly better

3. Falling asleep is unchanged

4. Falling asleep is slightly worse

5. Falling asleep is significantly worse

13. Have you noticed a change in the level of depressive symptoms while taking CBD/THC?

1. Depressive symptoms are significantly better

2. Depressive symptoms are slightly better

3. Depressive symptoms are unchanged

4. Depressive symptoms are slightly worse

5. Depressive symptoms are significantly worse

14. Have you noticed a difference in your short-term memory on days that you take your CBD/THC?

1. I have noticed severe short-term memory loss (forgetful >five times per day)

2. I have noticed moderate short-term memory loss (forgetful about three to five times per day)

3. I have noticed occasional short-term memory loss (forgetful about one to two times per day)

4. I have not noticed any changes in my short-term memory

5. My short-term memory has improved

15. Have you experienced feelings of “brain fog” or a diminishing ability to process information on days that you are taking your CBD/THC?

1. Brain fog is significantly worse

2. Brain fog is slightly worse

3. Brain fog is unchanged

4. Brain fog is slightly better

5. Brain fog is significantly better

6. I have not experienced brain fog

16. Do you notice a difference in how well-rested you feel in the mornings after taking your CBD/THC medication the night/day before?

1. I feel significantly less rested

2. I feel slightly less rested

3. I do not notice a difference in how rested I feel

4. I feel slightly more rested

5. I feel significantly more rested

17. Do you notice a difference in your balance after taking your CBD/THC medication?

1. My balance has been significantly improved

2. My balance has somewhat improved

3. My balance has not changed

4. My balance has been somewhat worse

5. My balance has been significantly worse

18. Do you notice a difference in your “freezing” after taking your CBD/THC medication?

1. My freezing has significantly improved

2. My balance has somewhat improved

3. My freezing has not changed

4. My freezing has been somewhat worse

5. My freezing has been significantly worse

6. I do not freeze regardless of the medication

19. Do you notice a difference in your rigidity after taking your CBD/THC medication?

1. My rigidity has significantly improved

2. My rigidity has somewhat improved

3. My rigidity has not changed

4. My rigidity has been somewhat worse

5. My rigidity has been significantly worse

6. I notice no rigidity regardless of the medication

20. How has your quality of life changed since taking your CBD/THC medication?

1. My quality of life has significantly improved

2. My quality of life has somewhat improved

3. My quality of life has not changed

4. My quality of life has somewhat worsened

5. My quality of life has significantly worsened

## References

[1] Goetz CG (2011). The history of Parkinson's disease: early clinical descriptions and neurological therapies. Cold Spring Harb Perspect Med.

[2] Marras C, Beck JC, Bower JH (2018). Prevalence of Parkinson's disease across North America. NPJ Parkinsons Dis.

[3] Radhakrishnan DM, Goyal V (2018). Parkinson's disease: a review. Neurol India.

[4] MacCallum CA, Russo EB (2018). Practical considerations in medical cannabis administration and dosing. Eur J Intern Med.

[5] Brunetti P, Pichini S, Pacifici R, Busardò FP, Del Rio A (2020). Herbal preparations of medical cannabis: a vademecum for prescribing doctors. Medicina (Kaunas).

[6] Bega D, Simuni T, Okun MS, Chen X, Schmidt P (2017). Medicinal cannabis for Parkinson’s disease: practices, beliefs, and attitudes among providers at National Parkinson Foundation Centers of Excellence. Mov Disord Clin Pract.

[7] Varshney K, Patel A, Ansari S, Shet P, Panag SS (2023). Cannabinoids in treating Parkinson’s disease symptoms: a systematic review of clinical studies. [PREPRINT]. Cannabis Cannabinoid Res.

[8] Lotan I, Treves TA, Roditi Y, Djaldetti R (2014). Cannabis (medical marijuana) treatment for motor and non-motor symptoms of Parkinson disease: an open-label observational study. Clin Neuropharmacol.

[9] Chagas MH, Zuardi AW, Tumas V (2014). Effects of cannabidiol in the treatment of patients with Parkinson's disease: an exploratory double-blind trial. J Psychopharmacol.

[10] Kindred JH, Li K, Ketelhut NB (2017). Cannabis use in people with Parkinson's disease and multiple sclerosis: a web-based investigation. Complement Ther Med.

[11] Urbi B, Corbett J, Hughes I (2022). Effects of cannabis in Parkinson’s disease: a systematic review and meta-analysis. J Parkinsons Dis.

[12] Nasreddine ZS, Phillips NA, Bédirian V (2005). The Montreal Cognitive Assessment, MoCA: a brief screening tool for mild cognitive impairment. J Am Geriatr Soc.

[13] Wechsler D (1997). WAIS-III: Administration and Scoring Manual: Wechsler Adult Intelligence Scale. https://www.worldcat.org/title/wais-iii-administration-and-scoring-manual-wechsler-adult-intelligence-scale/oclc/224913150.

[14] Hartlage L (1994). The Halstead-Reitan Neuropsychology Test Battery: Theory and Clinical Interpretation Second Edition: by Ralph M. Reitan, PhD & Deborah Wolfson, PhD, Tucson, AZ: Neuropsychology Press, 1993. Arch Clin Neuropsychol.

[15] Kaplan E, Goodglass H, Weintraub S (2016). American Psychological Association. Boston Naming Test. Boston Naming Test. PsycTESTS Dataset. Published online June.

[16] Vannorsdall TD, Maroof DA, Gordon B, Schretlen DJ (2012). Ideational fluency as a domain of human cognition. Neuropsychology.

[17] Benedict RHB, Schretlen D, Groninger L, Brandt J. Hopkins Verbal Learning Test--Revised (1991). American Psychological Association. Hopkins Verbal Learning Test--Revised. PsycTESTS Dataset. Published online.

[18] Proud EL, Miller KJ, Bilney B, Morris ME, McGinley JL (2020). Construct validity of the 9-Hole Peg Test and Purdue Pegboard Test in people with mild to moderately severe Parkinson's disease. Physiotherapy.

[19] Goetz CG, Tilley BC, Shaftman SR (2008). Movement Disorder Society-sponsored revision of the Unified Parkinson's Disease Rating Scale (MDS-UPDRS): scale presentation and clinimetric testing results. Mov Disord.

[20] Nowinski CJ, Siderowf A, Simuni T, Wortman C, Moy C, Cella D (2016). Neuro-QoL health-related quality of life measurement system: validation in Parkinson's disease. Mov Disord.

[21] Johns MW (1991). A new method for measuring daytime sleepiness: the Epworth sleepiness scale. Sleep.

[22] Stiasny-Kolster K, Mayer G, Schäfer S, Möller JC, Heinzel-Gutenbrunner M, Oertel WH (2007). The REM sleep behavior disorder screening questionnaire—a new diagnostic instrument. Mov Disord.

[23] Guerin JM, Copersino ML, Schretlen DJ (2018). Clinical utility of the 15-item Geriatric Depression Scale (GDS-15) for use with young and middle-aged adults. J Affect Disord.

[24] Schwab RS, England AC (1969). Projection Technique for Evaluating Surgery in Parkinson’s Disease. Third Symposium on Parkinson’s Disease.

[25] IBM Corp. Released 2020. IBM SPSS Statistics for Windows, Version 27.0. Armonk, NY: IBM Corp (2020). IBM Corp. IBM SPSS Statistics for Windows, version 27.0. https://www.ibm.com/support/pages/downloading-ibm-spss-statistics-27.

[26] Li G, Taljaard M, Van den Heuvel ER (2017). An introduction to multiplicity issues in clinical trials: the what, why, when and how. Int J Epidemiol.

[27] Arkell TR, Lintzeris N, Kevin RC (2019). Cannabidiol (CBD) content in vaporized cannabis does not prevent tetrahydrocannabinol (THC)-induced impairment of driving and cognition. Psychopharmacology (Berl).

[28] Finn DP, Haroutounian S, Hohmann AG, Krane E, Soliman N, Rice AS (2021). Cannabinoids, the endocannabinoid system, and pain: a review of preclinical studies. Pain.

[29] Thanabalasingam SJ, Ranjith B, Jackson R, Wijeratne DT (2021). Cannabis and its derivatives for the use of motor symptoms in Parkinson's disease: a systematic review and meta-analysis. Ther Adv Neurol Disord.

[30] de Almeida CM, Brito MM, Bosaipo NB (2021). Cannabidiol for rapid eye movement sleep behavior disorder. Mov Disord.

